# Metabolic syndrome severity is significantly associated with future coronary heart disease in Type 2 diabetes

**DOI:** 10.1186/s12933-017-0647-y

**Published:** 2018-01-19

**Authors:** Matthew J. Gurka, Yi Guo, Stephanie L. Filipp, Mark D. DeBoer

**Affiliations:** 1 Department of Health Outcomes and Policy, College of Medicine, University of Florida, Gainesville, FL 32608 USA; 2 Department of Pediatrics, Division of Pediatric Endocrinology, University of Virginia, 409 Lane Rd, Room 2017, P.O. Box 800386, Charlottesville, VA 22908 USA

**Keywords:** Cardiovascular disease, Diabetes, Metabolic syndrome, Risk

## Abstract

**Background:**

The severity of the metabolic syndrome (MetS) is significantly associated with future coronary heart disease (CHD) among individuals without baseline Type 2 diabetes. However, the validity of assessing MetS severity among individuals with diabetes is unknown.

**Objective:**

To assess for differences in MetS severity by timing of Type 2 diabetes diagnosis and to assess for associations between MetS severity and future CHD among individuals with diabetes.

**Methods:**

We analyzed data from participants of the Atherosclerosis Risk in Communities study, including 1419 with- and 7241 without diabetes, followed during 4 visits and adjudicated CHD diagnoses over a 20-year period. We used Cox-regression techniques to assess hazard ratios (HR) of CHD based on a sex- and race/ethnicity-specific MetS-severity Z-score (standard MetS score) and a similar MetS-severity score formulated without incorporating glucose as a component of MetS (no-glucose MetS score).

**Results:**

For both the standard- and no-glucose MetS-severity scores, scores were highest in the baseline-diabetes group, lowest in the never-diabetes group and intermediate in the incident-diabetes groups. Among participants with diabetes, increasing MetS-severity score at baseline was associated with incident CHD, using both the standard MetS score (HR 1.29, 95% confidence interval [CI] 1.21, 1.39) and the no-glucose score (HR 1.42, CI 1.24, 1.62) (both p < 0.001). For the baseline-diabetes group, this relationship remained significant when Visit 2 Hemoglobin-A_1c_ was included in the model, both for the standard MetS score (HR 1.21, CI 1.09, 1.34; p < 0.001) and the no-glucose score (HR 1.25, CI 1.04, 1.51; p = 0.02).

**Conclusions:**

MetS severity appears to provide an estimate of metabolic disarray in the setting of diabetes and is predictive of future CHD events beyond HbA1c. Identifying MetS severity among individuals with diabetes may help in identifying those at higher risk, who could then receive further preventative treatment.

**Electronic supplementary material:**

The online version of this article (10.1186/s12933-017-0647-y) contains supplementary material, which is available to authorized users.

## Introduction

Cardiovascular disease (CVD) remains the leading cause of mortality among patients with Type 2 diabetes mellitus (T2DM), continuing the need for predictive tools to identify those at highest risk [1]. The metabolic syndrome (MetS) is a cluster of cardiovascular risk factors that predicts future CVD in multiple settings [2–6], including among individuals with diabetes, among whom it carries hazard ratios (HR) ranging from 1.29 to 1.9 in males and 1.7 to 1.87 in females [7, 8]. MetS is composed of five individual components (central obesity, hypertension, hypertriglyceridemia, low HDL, and elevated fasting glucose) that are associated with insulin resistance and appear to be produced by underlying processes including cellular dysfunction, systemic inflammation, and oxidative stress [9–14]. However, use of traditional MetS criteria such as those of the Adult Treatment Panel (ATP)-III have been limited by their binary nature and by racial/ethnic discrepancies, underestimating risk among African Americans [15–18]. For these reasons we formulated a MetS-severity Z score that serves as a continuous estimate of metabolic derangement [19, 20]. Whereas ATP-III MetS has been criticized as “not being worth more than the sum of its parts” [21], this MetS-severity score when assessed among individuals without diabetes confers additional risk prediction for diabetes and coronary heart disease (CHD), even in models that include the individual MetS components [22, 23] —suggesting the potential that this score captures risk associated with the processes underlying MetS. We also developed a MetS-severity score that does not include glucose as a component and demonstrated that this no-glucose MetS score retained the ability to predict future diabetes [23].

These MetS-severity scores were derived and have been assessed only in populations without baseline diabetes. As such, it is unclear among individuals with diabetes whether assessing MetS severity would be an accurate means of tracking metabolic abnormalities over time and predicting future CHD. For the standard five-component MetS-severity score, it is possible that extreme fluctuations in fasting glucose may unduly influence the overall calculated score, lowering its predictive power. For the no-glucose score, it is possible that any CHD risk related to abnormalities in the other four MetS components would be inconsequential relative to the risk conferred by the degree of diabetes control.

Therefore, to evaluate the utility of a MetS-severity score among individuals with diabetes, our goals in the current study were (1) to compare MetS-severity levels by timing of diabetes diagnosis, (2) to assess for differences between the standard MetS-severity score and the no-glucose MetS score on a sex- and race-specific basis among individuals with diabetes, and (3) to evaluate correlations between these scores and future CHD among individuals with diabetes, both with and without adjustment for overall diabetes control, as assessed by hemoglobin A_1C_ (HbA_1c_). We hypothesized that these scores would continue to reflect metabolic abnormalities beyond glucose elevations alone and predict future CHD risk. These analyses hold relevance for the establishing the validity of assessing MetS severity as a marker of CHD risk in the setting of diabetes.

## Methods

### Study population

The Atherosclerosis Risk in Communities (ARIC) study is a large community-based epidemiological cohort study across 4 field centers in the US, with timing as follows: Visit 1 (1987–1989), Visit 2 (1990–1992), Visit 3 (1993–1995) and Visit 4 (1996–1998), and on-going follow-up for adjudicated CHD outcomes thereafter. This study and/or its analysis was approved by the Institutional Review Boards of the University of Florida, the University of Virginia, and the study sites for ARIC and was carried out in accordance with the Declaration of Helsinki. ARIC data are available through ancillary study collaboration with ARIC [24]. A total of 15,397 participants aged 45–64 years provided informed consent to be included in the study—5359 white men, 5943 white women, 1585 African-American men, and 2464 African-American women and 46 participants of other races. Further details of the study design and objectives are published elsewhere [24]. From this sample, we excluded those who reported presence of CHD at the baseline visit (n = 747), who missed any of the follow-up visits (n = 4511), participants other than African Americans and whites (n = 46), or those with missing data on any of the components of MetS (n = 4821). Thus, a total of 8660 participants were included in the current analyses.

### Measurement of metabolic syndrome components

Previous reports have published details of procedures for blood collection and analysis for lipids [25] and serum glucose [26]. Briefly, participants fasted overnight for 12 h before the examination. Phlebotomy was performed, blood sample was centrifuged and serum was sent to a central laboratory for examination. Triglycerides were measured by enzymatic methods, and HDL cholesterol was measured after dextran-magnesium precipitation. LDL cholesterol was calculated using the Friedewald equation. Serum glucose was measured by the hexokinase-6 phosphate dehydrogenase method [27]. HbA_1c_ was measured only at Visit 2 via high-performance liquid chromatography using the Tosoh A1c-2.2 Plus Glycohemoglobin Analyzer or the Tosoh G7 (Tosoh Corp., Tokyo, Japan), with both instruments standardized to the Diabetes Control and Complications Trial assay [28]. Trained clinical staff measured waist circumference at the umbilical level to the nearest cm. BP was examined in sitting position with a random-zero sphygmomanometer—of the three measurements performed, the average of the last two measurements were used for analysis. Similar procedures were followed at all study sites over the 4 visits.

### Assessment of covariates and outcomes

#### MetS severity

MetS severity was calculated as a Z-score for participants at all four visits using sex- and race- based formulae. As described elsewhere [19, 20], these scores were derived using a confirmatory factor analysis approach for the five traditional MetS components (WC, triglycerides, HDL-cholesterol, systolic BP, fasting glucose). This common statistical approach determines the degree to which of each of the five components contributes to a latent MetS “factor”. We performed this confirmatory factor analysis among adults age 20–64 years from the National Health and Nutrition Examination Survey (NHANES) with categorization into six sub-groups based on sex and race/ethnicity (non-Hispanic white, non-Hispanic black and Hispanic). For each of these six population sub-groups, the loading coefficients for the five MetS components (the degree of contribution to the latent MetS factor) were transformed into a single MetS factor were then used to generate equations to calculate a standardized MetS severity score for each sub-group (http://mets.health-outcomes-policy.ufl.edu/calculator/). The resulting MetS severity scores in theory operate as Z-scores (normally distributed and ranging from theoretical negative to positive infinity with mean = 0 and SD = 1) of relative MetS severity on a sex- and race/ethnicity-specific basis. This score can be followed over time [29, 30] and correlates with other markers of MetS risk, including hsCRP, uric acid and the homeostasis model of insulin resistance, with adiponectin [20, 31]. Among individuals without baseline diabetes, these scores were correlated with long-term CVD and T2DM risk [22, 23, 31–33]. We separately derived a set of scores that utilized only measures of WC, BP, triglycerides and HDL (referred to as “no-glucose” score) [23]. Among individuals without baseline diabetes, these scores were associated with increased risk of diabetes [23].

Traditional MetS was defined using the ATP-III criteria, i.e. presence of three or more of the following abnormalities: elevated waist circumference (≥ 102 cm for men, ≥ 88 cm for women), elevated triglycerides (1.69 mmol/L [≥ 150 mg/dL] or drug treatment for elevated triglycerides), reduced HDL (1.03 mmol/L [< 40 mg/dL] for men, 1.29 mmol/L [< 50 mg/dL] for women or drug treatment for reduced HDL), elevated BP (≥ 130 mmHg systolic or ≥ 85 mmHg diastolic or drug treatment for hypertension) and elevated blood glucose (≥ 5.6 mmol/L [≥ 100 mg/dl] or drug treatment for elevated glucose) [9].

#### Diabetes definition

Participants were defined as having diabetes if they reported that a physician had told them they had diabetes, had a fasting glucose ≥ 7 mmol/L (≥ 126 mg/dL) or a non-fasting glucose ≥ 11.1 mmol/L (≥ 200 mg/dL), or if they reported they were taking insulin or oral hypoglycemic medications [34, 35].

#### Incident CHD

Incident CHD was ascertained using standard ARIC protocols [36, 37] and included fatal or nonfatal hospitalized myocardial infarction, fatal CHD, silent myocardial infarction identified by electrocardiography, or coronary revascularization. Follow-up time for incident CHD events was the minimum number of days between the baseline visit and either the first event, death from other causes, last contact, or Dec 31, 2011 [36, 37].

#### Statistics

We assessed the ability of the MetS-severity score (both with and without glucose) at the time of the visit where diabetes was identified to predict future CHD in Cox proportional-hazards models, using the Fine-Gray approach to account for competing risk of non-CHD death. These models were stratified by site and included age (at baseline), sex, and race as covariates. Interactions between MetS severity and sex and race were initially examined; if interactions were not significant they were removed from the model. These models were compared to a model that included ATP-III MetS as the primary predictor, and a model that included the number of ATP-III MetS elevated components (ranging from 0 to 5 as another potential measure of MetS severity). Model fit was compared between the four models by the Akaike Information Criterion (AIC), with smaller values indicating a better fit. As diabetes could have developed at any one of four visits (present at Visit 1, or developed by Visits 2, 3, and 4), we also examined MetS HR’s across the four different diabetes groups. In all cases, we did not include individuals who developed CHD before diabetes. Additionally, to examine the confounding influence of HbA_1c_, models were fit that included Visit 2 HbA_1c_ (the only visit it was available), only for those who had diabetes at Visit 1. Hazard ratios (HR) and 95% confidence intervals, as well as model-estimated cumulative incidence of CHD over time, were calculated and of primary interest.

## Results

### Demographics

Table 1 displays age, sex and race of participants for each of the groups (never-diabetes, baseline-diabetes, and incident-diabetes by visit number). Age decreased by group, with the baseline-diabetes group being oldest (55.2 years), those in the incident diabetes groups being gradually younger, and those in the never-diabetes group being youngest (53.8 years). There was a greater percentage of males and black participants in the baseline and incident diabetes groups compared to the never-diabetes group.

**Table 1:** Participant characteristics (frequency (%) unless otherwise noted), overall and by diabetes status

	Overall (n = 8660)	Diabetes status				
		Never diabetes (n = 7241)	Baseline diabetes (n = 536)	Incident diabetes by:		
				Visit 2 (n = 375)	Visit 3 (n = 260)	Visit 4 (n = 248)
Baseline age (mean (SD))	53.89 (5.65)	53.79 (5.65)	55.20 (5.76)	54.40 (5.68)	53.66 (5.59)	53.61 (5.17)
Sex						
Female	4873 (56.3%)	4150 (57.3%)	277 (51.7%)	200 (53.3%)	126 (48.5%)	120 (48.4%)
Male	3787 (43.7%)	3091 (42.7%)	259 (48.3%)	175 (46.7%)	134 (51.5%)	128 (51.6%)
Race						
Black	1464 (16.9%)	1121 (15.5%)	115 (21.5%)	104 (27.7%)	61 (23.5%)	63 (25.4%)
White	7196 (83.1%)	6120 (84.5%)	421 (78.5%)	271 (72.3%)	199 (76.5%)	185 (74.6%)
ATP-III MetS at baseline	2967 (34.26%)	1952 (26.96%)	415 (77.43%)	267 (71.20%)	168 (64.62%)	165 (66.53%)
Baseline values (mean (SD)):						
MetS z-score	0.16 (0.91)	− 0.03 (0.73)	1.75 (1.42)	0.78 (0.69)	0.67 (0.69)	0.66 (0.64)
MetS score (no glucose)	0.05 (0.77)	− 0.05 (0.74)	0.72 (0.79)	0.51 (0.73)	0.47 (0.68)	0.51 (0.69)
Waist circumference	95.80 (13.31)	94.03 (12.51)	105.88 (14.00)	104.87 (13.43)	103.18 (13.74)	104.45 (12.57)
Systolic blood pressure	120.75 (18.31)	119.19 (17.81)	130.40 (18.84)	127.99 (18.50)	128.26 (19.68)	126.42 (17.86)
Triglycerides	126.41 (80.57)	118.65 (71.06)	188.47 (137.08)	153.58 (87.71)	148.48 (78.41)	154.66 (90.77)
HDL-cholesterol	52.48 (16.71)	53.89 (16.87)	44.44 (13.60)	46.29 (14.03)	45.62 (13.12)	45.23 (14.40)
Fasting glucose	101.94 (22.17)	97.05 (8.20)	158.16 (57.76)	111.02 (9.19)	107.40 (9.62)	103.92 (8.91)
Follow-up time (years) since diabetes diagnosis (mean(SD))^a^	20.17 (5.31)	20.98 (4.77)	17.98 (6.31)	16.79 (5.26)	14.18 (4.97)	11.94 (4.03)
Incident CHD during follow-up	1446 (16.70%)	1049 (14.49%)	187 (34.89%)	88 (23.47%)	61 (23.46%)	61 (24.60%)

^a^ Visit 1 for those in the Never Diabetes group

### MetS-severity scores by timing of diabetes diagnosis

**Fig. 1:**
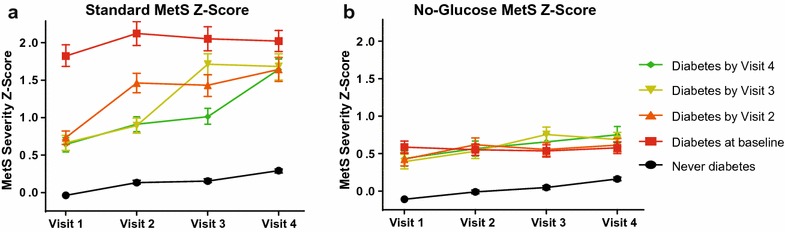
Standard- and no-glucose MetS severity Z-scores by timing of diabetes diagnosis. Model-generated values for **a** standard and **b** no-glucose MetS severity Z-scores for participants with diabetes at baseline (Visit 1), and those diagnosed by Visits 2, 3, and 4, compared to those never diagnosed. All models were stratified by site and included age (at baseline), sex, and race as covariates

Figure 1 provides (A) standard 5-component and (B) no-glucose MetS-severity scores by timing of diabetes diagnosis. Compared to all other groups, participants with baseline diabetes had the highest standard MetS-severity scores at baseline and thereafter, while those in the never-diabetes group had levels that remained low through all visits (though with a gradual increase over time). Participants with incident diabetes at Visits 2–4 had mean standard MetS-severity scores that were similar to each other at baseline but that exhibited a sharp increase in score at the visit associated with diabetes diagnosis and continued elevated thereafter. By Visit 4 these incident-diabetes groups converged to similar mean scores.

In assessing the no-glucose score, the baseline-diabetes group had MetS-severity Z-scores that were much lower than seen when using the standard MetS-severity score (0.59 vs. 1.82). The baseline-diabetes group had mean no-glucose scores that were highest of all groups at baseline but that were similar to the incident-diabetes groups at subsequent visits. The incident-diabetes groups exhibited no-glucose scores that (1) were similar to each other at baseline; (2) were only modestly different at baseline from their standard MetS scores (0.4 vs. 0.7); and (3) exhibited a significant (p < 0.01) increase in score at the visit corresponding with diabetes diagnosis. Those in the never-diagnosis group exhibited scores that were low and not appreciably different from their standard scores.

Additional file 1: Figure S1 displays the individual MetS components by timing of diabetes diagnosis. Levels for each component were lowest in the never-diabetes group. At baseline, levels of triglycerides and fasting glucose were highest in the baseline-diabetes group, with overall similar levels of the individual components between the baseline- and incident diabetes groups.

### MetS-severity scores by sex and race

**Fig. 2:**
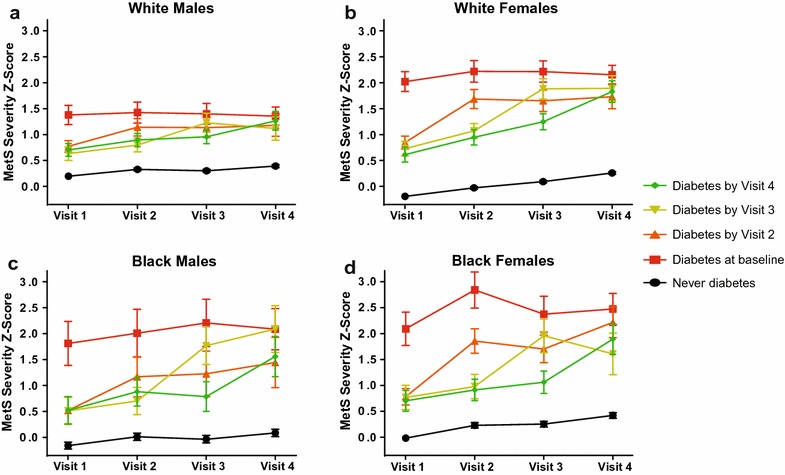
Standard MetS severity Z-scores by sex and race. Model-generated values of MetS severity for **a** white males, **b** white females, **c** black males, and **d** black females for participants with diabetes at baseline (Visit 1), and those diagnosed by Visits 2, 3, and 4, compared to those never diagnosed. All models were stratified by site and included age at baseline as a covariate

Figure 2 and Additional file 1: Figure S2 display standard- and no-glucose MetS-severity scores (respectively) by sex and race. In evaluating the standard MetS-severity score, each of the sex and race subgroups had scores and patterns that in general were similar to those for the overall group and to each other. The exceptions were: (1) white males, for whom mean MetS-severity scores were lower than the overall group among those with baseline diabetes and higher than the overall group among the never-diabetes group, resulting in a narrower range of scores; and (2) black males and females, who were the only groups to exhibit an increase in MetS severity in visits following diabetes diagnosis; this was true for black males with incident diabetes at Visits 2 and 3 and black females with incident diabetes at Visits 2, later increasing by Visit 4.

For the no-glucose scores, white males and females had higher scores than black males and females; the exception to this was in the never-diabetes group, in which white- and black females had scores that were similar to each other. For all sex- and race subgroups, each incident-diabetes group exhibited its highest no-glucose MetS scores at the visit corresponding to diabetes diagnosis. Elevations in no-glucose MetS severity were associated with higher fasting glucose levels at all time points (p < 0.0001).

### CHD risk by MetS-severity score

**Fig. 3:**
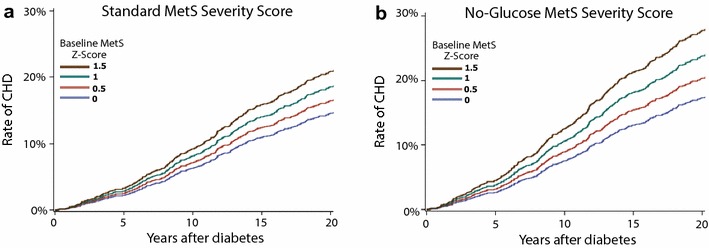
Standard MetS severity Z-scores by sex and race. Cox-regression analysis of time from diabetes diagnosis to diagnosis of coronary heart disease (CHD) for participants with **a** standard and **b** no-glucose MetS severity Z-scores of 0, 0.5, 1 and 1.5. Models were stratified by site and included age (at baseline), sex, and race as covariates

Among participants with diabetes there were 1446 incident cases of CHD, representing 16.7% of the cohort (Table 1). Additional file 2: Table S1 provides results of models for incident CHD by MetS-severity scores, adjusted for baseline age, sex and race. Risk of CHD was more common with rising age, among males vs. females and among whites vs. blacks. Using the standard MetS-severity score, each 1-SD unit increase in MetS severity was associated with a HR for future CHD of 1.29 (95% confidence interval [CI] 1.21, 1.39), while the no-glucose MetS severity score had a HR of 1.42 (CI 1.24, 1.62; all p < 0.001). There did not appear to be a difference by sex or racial/ethnic group in these relationships. These HR’s compared to participants in the never-diabetes group that had HR of 1.49 (CI 1.37, 1.62) using the standard MetS score and 1.53 (CI 1.41, 1.66) using the no-glucose score (both p < 0.001). When comparing the AIC values across the four models using the four different measures of MetS (MetS severity, no-glucose MetS severity, ATP-III MetS, and number of ATP-III MetS elevations), the AIC values from the two MetS severity scores were the smallest, indicating the best model fit. Figure 3 displays cumulative incidence of CHD by MetS-severity score, generated from the models in Additional file 2: Table S1, with an increase in incidence with rising MetS-severity score at diabetes diagnosis.

In separating the estimation of the hazard ratios across the four groups of individuals defined by when diabetes was diagnosed (Visits 1, 2, 3, 4), no statistical differences were observed when comparing the HR’s by time of diagnosis (Additional file 2: Table S2). Lack of statistical power may have contributed to not observing significant interactions between any of the MetS measures and time of diabetes diagnosis.

Among individuals with baseline diabetes, these relationships between MetS severity and CHD risk did not appear to be heavily affected by the degree of diabetes control as estimated by Visit 2 HbA_1c_ (Additional file 2: Table S3), with HR’s that decreased from 1.24 (CI 1.3, 1.36) to 1.21 using the standard MetS-severity score and from 1.34 (CI 1.12, 1.60) to 1.25 (CI 1.04, 1.51) using the no-glucose score.

## Discussion

We found that a continuous estimate of MetS severity retained the ability to predict CHD risk among individuals with Type 2 diabetes. This was the case despite the potential for elevations in glucose to unduly influence MetS-severity score levels. While excursions in fasting glucose levels were clearly reflected in higher MetS severity scores at diagnosis—with an increase in Z-scores of approximately 0.7 SD units—these did not appear to affect the relationship between the score and future CHD in that MetS severity provided overall similar risk prediction when using the standard MetS severity score as when using a score derived without glucose as a component. Additionally, higher MetS severity scores among individuals with diabetes did not appear to be merely an estimate of overall diabetes control in that the relationships between MetS severity and future CHD remained following adjustment for HbA_1c_. In contrast to scoring systems specifically designed to predict CHD in Type 2 diabetes [38], this MetS severity score was designed to estimate MetS severity—potentially as a means of estimating the processes behind MetS [9–11]. As such, this increase in risk may represent risk conferred by underlying oxidative stress and inflammation in addition to additive risk from the individual MetS components. Overall, these data provide evidence for the potential utility for tracking MetS severity among individuals with diabetes as a means of identifying those at highest risk for CHD, for whom higher-intensity preventative measures could be taken.

We noted that in the incident-diabetes groups MetS severity scores were already elevated up to 10 years before diabetes diagnosis—which is not surprising, given that these scores are correlated with risk of developing diabetes [23, 33]. These elevations appeared to be due to abnormalities in each of the components of MetS (Additional file 1: Figure S1), revealing that a major difference between the incident-diabetes and never-diabetes groups was a consistent background level of MetS severity related to obesity and potentially a genetic propensity toward the processes underlying insulin resistance [39].

The increase in MetS severity scores at the visit corresponding to diabetes diagnosis—with a near doubling of the difference from the never-diabetes group—appeared to be due mostly to changes in glucose, as the non-glucose score did not exhibit as drastic of fluctuations. This suggested against severe effects of glucose elevations on the pathological processes that underlie MetS and produce abnormalities in the other components. Following this increase in score at diabetes diagnosis, the standard MetS-severity score remained overall stable (potentially reflecting ongoing challenges with glucose levels) while the no-glucose score actually decreased slightly (likely reflecting effects of treatment of initial glucose and possibly other abnormalities). Those with baseline diabetes (of potential longstanding duration) maintained higher standard MetS-severity scores compared to all other groups throughout the study. This also appeared to primarily reflect abnormalities in glucose alone, since there did not appear to be meaningful differences between diabetes groups by time of diagnosis when using the no-glucose MetS score.

In considering racial/ethnic differences, we noted that white males in the baseline-diabetes group had scores that were significantly lower than other subgroups by sex and race, while white males in the never-diabetes group had the highest scores of all the subgroups. This resulted in a gap in MetS severity between those with and without diabetes was narrowest among white males, suggesting that compared to other groups, white males exhibit less metabolic heterogeneity in metabolic risk. In contrast to white participants, who experienced a decrease or maintenance of standard 5-component MetS severity scores, black males and females frequently experienced an increase in MetS severity scores in the visits after diabetes diagnosis. It is unclear whether this reflects differences in access to medical treatments [40] or relates to the overall severity of the underlying diabetes.

CHD risk related to MetS severity was slightly lower among individuals with diabetes relative to those without diabetes, with HR’s of 1.29 vs. 1.49 for standard MetS-severity score and 1.42 vs. 1.53 for the no-glucose score, likely related to the elevated rate of CHD overall in the setting of diabetes [1, 22]. Nevertheless, the consistent nature of these relationships suggests that processes related to MetS and its components are involved in on-going contributions to CHD risk, even in the setting of diabetes. This may relate either to separate roles for abnormalities in each of the components [41–43] (risk that is then integrated into the MetS severity score) or risk related to the pathophysiological processes underlying MetS (as had been suggested by the associations between CHD and MetS severity, independent of its components [22, 23]). Either way, the association of MetS severity and CHD risk raises the potential utility to following MetS severity over time among individuals with diabetes, targeting a lower score to reduce risk of future CVD.

This study had multiple limitations, including that the timing of diabetes diagnosis was limited to study visits; this was unlike CHD diagnosis, which was assigned using an adjudication protocol based on records from hospitalization and deaths. Additionally, we lacked details regarding the timing and adherence of treatment of diabetes—which clearly affected fasting glucose levels, as well as overall diabetes control and metabolic disarray. We had HbA_1c_ levels only at Visit 2, limiting our analysis of how overall diabetes control affected the relationship between MetS severity and CHD to those with diabetes at baseline. Finally, this study began in an era prior to the availability of statin medications, limiting generalizability to the current population of individuals with diabetes. However, this study also had multiple strengths, including assessment in a large cohort with long-term adjudicated CHD outcomes.

## Conclusions

These sex- and race/ethnicity-specific MetS-severity Z-scores appear to provide a valid estimate of metabolic derangement and CHD risk among individuals with diabetes. Scores such as these could be tracked over time in patients with diabetes as an integrated estimate of CHD risk, potentially as a motivator for individuals with diabetes and their physicians toward risk reduction.

## Additional files

**Additional file 1: Figure S1.** Levels of the individual MetS components by timing of diabetes diagnosis. Model-generated values of A). waist circumference, B). systolic blood pressure, C). HDL-cholesterol, D). fasting triglycerides, and E). fasting glucose for participants with diabetes at baseline (Visit 1), and those diagnosed by Visits 2, 3, and 4, compared to those never diagnosed. All models were stratified by site and included age (at baseline), sex, and race as covariates. **Figure S2.** No-glucose MetS severity Z-scores by sex and race. Model-generated values for A). standard and B). no-glucose MetS severity Z-scores for participants with diabetes at baseline (Visit 1), and those diagnosed by Visits 2, 3, and 4, compared to those never diagnosed. All models were stratified by site and included age (at baseline), sex, and race as covariates.

**Additional file 2: Table S1.** Models of MetS (and MetS severity) in individuals with diabetes on incident CHD. **Table S2.** Models of MetS (and MetS severity) in individuals with diabetes on incident CHD by time of diabetes diagnosis. **Table S3.** Models of standard MetS severity in individuals with baseline diabetes on incident CHD with and without inclusion of HbA1c at Visit 2.

## Abbreviations

ARIC: Atherosclerosis Risk in Communities
ATP-III: Adult Treatment Panel III
CVD: cardiovascular disease
MetS: metabolic syndrome
NHANES: National Health and Nutrition Examination Survey

## References

[1] Benjamin EJ, Blaha MJ, Chiuve SE, Cushman M, Das SR, Deo R, de Ferranti SD, Floyd J, Fornage M, Gillespie C (2017). Heart disease and stroke statistics—2017 update: a report from the American Heart Association. Circulation.

[2] van Herpt TT, Dehghan A, van Hoek M, Ikram MA, Hofman A, Sijbrands EJ, Franco OH (2016). The clinical value of metabolic syndrome and risks of cardiometabolic events and mortality in the elderly: the Rotterdam Study. Cardiovasc Diabetol.

[3] Kurl S, Laaksonen DE, Jae SY, Mäkikallio TH, Zaccardi F, Kauhanen J, Ronkainen K, Laukkanen JA (2016). Metabolic syndrome and the risk of sudden cardiac death in middle-aged men. Int J Cardiol.

[4] Xanthakis V, Sung JH, Samdarshi TE, Hill AN, Musani SK, Sims M, Ghraibeh KA, Liebson PR, Taylor HA, Vasan RS (2015). Relations between subclinical disease markers and type 2 diabetes, metabolic syndrome, and incident cardiovascular disease: the Jackson Heart Study. Diabetes Care.

[5] Younis A, Tzur B, Peled Y, Shlomo N, Goldenberg I, Fisman EZ, Tenenbaum A, Klempfner R (2016). Metabolic syndrome is independently associated with increased 20-year mortality in patients with stable coronary artery disease. Cardiovasc Diabetol.

[6] Vinluan CM, Zreikat HH, Levy JR, Cheang KI (2012). Comparison of different metabolic syndrome definitions and risks of incident cardiovascular events in the elderly. Metabolism.

[7] Guzder RN, Gatling W, Mullee MA, Byrne CD (2006). Impact of metabolic syndrome criteria on cardiovascular disease risk in people with newly diagnosed type 2 diabetes. Diabetologia.

[8] Sone H, Mizuno S, Fujii H, Yoshimura Y, Yamasaki Y, Ishibashi S, Katayama S, Saito Y, Ito H, Ohashi Y (2005). Is the diagnosis of metabolic syndrome useful for predicting cardiovascular disease in asian diabetic patients? Analysis from the Japan Diabetes Complications Study. Diabetes Care.

[9] Grundy SM, Cleeman JI, Daniels SR, Donato KA, Eckel RH, Franklin BA, Gordon DJ, Krauss RM, Savage PJ, Smith SC (2005). Diagnosis and management of the metabolic syndrome—An American Heart Association/National Heart, Lung, and Blood Institute Scientific Statement. Circulation.

[10] Shulman GI (2014). Ectopic fat in insulin resistance, dyslipidemia, and cardiometabolic disease. N Engl J Med.

[11] DeBoer MD (2013). Obesity, systemic inflammation, and increased risk for cardiovascular disease and diabetes among adolescents: a need for screening tools to target interventions. Nutrition.

[12] Abraham TM, Pedley A, Massaro JM, Hoffmann U, Fox CS (2015). Association between visceral and subcutaneous adipose depots and incident cardiovascular disease risk factors. Circulation.

[13] Zethelius B, Cederholm J (2015). Comparison between indexes of insulin resistance for risk prediction of cardiovascular diseases or development of diabetes. Diabetes Res Clin Pract.

[14] Hulten EA, Bittencourt MS, Preston R, Singh A, Romagnolli C, Ghoshhajra B, Shah R, Abbasi S, Abbara S, Nasir K (2017). Obesity, metabolic syndrome and cardiovascular prognosis: from the Partners coronary computed tomography angiography registry. Cardiovasc Diabetol.

[15] Walker SE, Gurka MJ, Oliver MN, Johns DW, DeBoer MD (2012). Racial/ethnic discrepancies in the metabolic syndrome begin in childhood and persist after adjustment for environmental factors. Nutr Metab Cardiovasc Dis.

[16] DeBoer MD, Gurka MJ, Sumner AE (2011). Diagnosis of the metabolic syndrome is associated with disproportionately high levels of high-sensitivity C-reactive protein in non-hispanic black adolescents: an analysis of NHANES 1999–2008. Diabetes Care.

[17] DeBoer MD, Dong L, Gurka MJ (2011). Racial/ethnic and sex differences in the ability of metabolic syndrome criteria to predict elevations in fasting insulin levels in adolescents. J Pediatr.

[18] DeBoer MD, Dong L, Gurka MJ (2012). Racial/ethnic and sex differences in the relationship between uric acid and metabolic syndrome in adolescents: an analysis of National Health and Nutrition Survey 1999-2006. Metabolism.

[19] Gurka MJ, Ice CL, Sun SS, DeBoer MD (2012). A confirmatory factor analysis of the metabolic syndrome in adolescents: an examination of sex and racial/ethnic differences. Cardiovasc Diabetol.

[20] Gurka MJ, Lilly CL, Norman OM, DeBoer MD (2014). An examination of sex and racial/ethnic differences in the metabolic syndrome among adults: a Confirmatory Factor Analysis and a Resulting Continuous Severity Score. Metabolism.

[21] Reaven GM (2011). The metabolic syndrome: time to get off the merry-go-round?. J Intern Med.

[22] DeBoer MD, Gurka MJ, Hill Golden S, Musani SK, Sims M, Vishnu A, Guo Y, Pearson TA (2017). Independent associations between metabolic syndrome severity & future coronary heart disease by sex and race. J Am Coll Card.

[23] Gurka MJ, Golden SH, Musani SK, Sims M, Vishnu A, Guo Y, Cardel M, Pearson TA, DeBoer MD (2017). Independent associations between a metabolic syndrome severity score and future diabetes by sex and race: the Atherosclerosis Risk In Communities Study and Jackson Heart Study. Diabetologia.

[24] The Atherosclerosis Risk in Communities (1989). (ARIC) Study: design and objectives. The ARIC investigators. Am J Epidemiol.

[25] Sharrett AR, Ballantyne CM, Coady SA, Heiss G, Sorlie PD, Catellier D, Patsch W (2001). Atherosclerosis Risk in Communities Study G: coronary heart disease prediction from lipoprotein cholesterol levels, triglycerides, lipoprotein(a), apolipoproteins A-I and B, and HDL density subfractions: The Atherosclerosis Risk in Communities (ARIC) Study. Circulation.

[26] Folsom AR, Szklo M, Stevens J, Liao F, Smith R, Eckfeldt JH (1997). A prospective study of coronary heart disease in relation to fasting insulin, glucose, and diabetes. The Atherosclerosis Risk in Communities (ARIC) Study. Diabetes Care.

[27] McNeill AM, Schmidt MI, Rosamond WD, East HE, Girman CJ, Ballantyne CM, Golden SH, Heiss G (2005). The metabolic syndrome and 11-year risk of incident cardiovascular disease in the atherosclerosis risk in communities study. Diabetes Care.

[28] Selvin E, Steffes MW, Zhu H, Matsushita K, Wagenknecht L, Pankow J, Coresh J, Brancati FL (2010). Glycated hemoglobin, diabetes, and cardiovascular risk in nondiabetic adults. N Engl J Med.

[29] Vishnu A, Gurka MJ, DeBoer MD (2015). The severity of the metabolic syndrome increases over time within individuals, independent of baseline metabolic syndrome status and medication use: the Atherosclerosis Risk in Communities Study. Atherosclerosis.

[30] Gurka MJ, Vishnu A, Santen RJ, DeBoer MD: Progression of Metabolic Syndrome Severity During the Menopausal Transition. *J Am Heart Assoc* 2016, 5(8). 10.1161/JAHA.116.003609 PMC5015287 27487829

[31] DeBoer MD, Gurka MJ, Morrison JA, Woo JG (2016). Inter-relationships between the severity of metabolic syndrome, insulin and adiponectin and their relationship to future type 2 diabetes and cardiovascular disease. Int J Obes (Lond).

[32] DeBoer MD, Gurka MJ, Woo JG, Morrison JA (2015). Severity of Metabolic Syndrome as a Predictor of Cardiovascular Disease Between Childhood and Adulthood: the Princeton Lipid Research Cohort Study. J Am Coll Cardiol.

[33] DeBoer MD, Gurka MJ, Woo JG, Morrison JA (2015). Severity of the metabolic syndrome as a predictor of type 2 diabetes between childhood and adulthood: the Princeton Lipid Research Cohort Study. Diabetologia.

[34] Schmidt MI, Duncan BB, Bang H, Pankow JS, Ballantyne CM, Golden SH, Folsom AR, Chambless LE (2005). Investigators ARiC: identifying individuals at high risk for diabetes: The Atherosclerosis Risk in Communities study. Diabetes Care.

[35] Effoe VS, Correa A, Chen H, Lacy ME, Bertoni AG (2015). High-Sensitivity C-Reactive Protein Is Associated With Incident Type 2 Diabetes Among African Americans: the Jackson Heart Study. Diabetes Care.

[36] Folsom AR, Wu KK, Rosamond WD, Sharrett AR, Chambless LE (1997). Prospective study of hemostatic factors and incidence of coronary heart disease: the Atherosclerosis Risk in Communities (ARIC) Study. Circulation.

[37] Keku E, Rosamond W, Taylor HA, Garrison R, Wyatt SB, Richard M, Jenkins B, Reeves L, Sarpong D (2005). Cardiovascular disease event classification in the Jackson Heart Study: methods and procedures. Ethn Dis.

[38] Stevens RJ, Kothari V, Adler AI, Stratton IM (2001). Group UKPDSU: the UKPDS risk engine: a model for the risk of coronary heart disease in Type II diabetes (UKPDS 56). Clin Sci (Lond).

[39] Dupuis J, Langenberg C, Prokopenko I, Saxena R, Soranzo N, Jackson AU, Wheeler E, Glazer NL, Bouatia-Naji N, Gloyn AL (2010). New genetic loci implicated in fasting glucose homeostasis and their impact on type 2 diabetes risk. Nat Genet.

[40] Association AD (2017). 1. Promoting Health and Reducing Disparities in Populations. Diabetes Care.

[41] Sowers JR, Epstein M, Frohlich ED (2001). Diabetes, hypertension, and cardiovascular disease: an update. Hypertension.

[42] Cullen P (2000). Evidence that triglycerides are an independent coronary heart disease risk factor. Am J Cardiol.

[43] Després JP, Lemieux I, Dagenais GR, Cantin B, Lamarche B (2000). HDL-cholesterol as a marker of coronary heart disease risk: the Québec cardiovascular study. Atherosclerosis.

