# Supplementary material for: Metabolic syndrome severity is significantly associated with future coronary heart disease in Type 2 diabetes
Source: Cardiovasc Diabetol. 2018 Jan 19;17:17. doi: 10.1186/s12933-017-0647-y (PMC5775549; doi:10.1186/s12933-017-0647-y)
Supplement: Supplementary file 2 — Additional file 2: Table S1. Models of MetS (and MetS severity) in individuals with diabetes on incident CHD. Table S2. Models of MetS (and MetS severity) in individuals with diabetes on incident CHD by time of diabetes diagnosis. Table S3. Models of standard MetS severity in individuals with baseline diabetes on incident CHD with and without inclusion of HbA1c at Visit 2. [file 12933_2017_647_MOESM2_ESM.docx]

**Table S1: Models of MetS (and Severity) in Individuals with Diabetes on Incident CHD**

Using MetS Severity with Glucose, the No-Glucose MetS Severity Score, ATP-III MetS Classification, and Number of ATP-III MetS Components at Time of Diagnosis

| Model Covariate | Model including  MetS Severity | |  | Model including  No-Glucose MetS Severity | |  | Model Including  ATP-III MetS | |  | Model including Number of  ATP-III MetS Components | |
| --- | --- | --- | --- | --- | --- | --- | --- | --- | --- | --- | --- |
|  | Hazard Ratio (95% CI) | p-value |  | Hazard Ratio (95% CI) | p-value |  | Hazard Ratio (95% CI) | p-value |  | Hazard Ratio (95% CI) | p-value |
|  |  |  |  |  |  |  |  |  |  |  |  |
| Baseline Age | 1.03 (1.01, 1.04) | < 0.001 |  | 1.03 (1.01, 1.04) | < 0.001 |  | 1.02 (1.00, 1.04) | 0.016 |  | 1.02 (1.00, 1.04) | 0.017 |
|  |  |  |  |  |  |  |  |  |  |  |  |
| Male (vs. Female) | 1.97 (1.58, 2.46) | < 0.001 |  | 1.74 (1.41, 2.14) | < 0.001 |  | 1.69 (1.37, 2.09) | < 0.001 |  | 1.76 (1.43, 2.17) | < 0.001 |
|  |  |  |  |  |  |  |  |  |  |  |  |
| Black (vs. White) | 0.36 (0.15, 0.85) | 0.020 |  | 0.44 (0.19, 1.04) | 0.061 |  | 0.38 (0.16, 0.90) | 0.027 |  | 0.41 (1.13, 1.34) | 0.038 |
|  |  |  |  |  |  |  |  |  |  |  |  |
| MetS Measure  (severity or ATP-III  status per column heading)  at time of diabetes diagnosis | 1.29 (1.21, 1.39) | <0.001 |  | 1.42 (1.24, 1.62) | < 0.001 |  | 1.61 (1.21, 2.14) | 0.001 |  | 1.23 (1.13, 1.34) | < 0.001 |
|  |  |  |  |  |  |  |  |  |  |  |  |
| *Model AIC* | *4163.19* |  |  | *4175.07* |  |  | *4188.41* |  |  | *4179.75* |  |
|  |  |  |  |  |  |  |  |  |  |  |  |

**Table S2: Models of MetS (and Severity) in Individuals with Diabetes on Incident CHD**

**By Time of Diabetes Diagnosis**

Using MetS Severity with Glucose, the No-Glucose MetS Severity Score, ATP-III MetS Classification, and Number of ATP-III MetS Components at Time of Diagnosis

| MetS Measure  (Severity or ATP-III  Status per Column Heading)  At Time of Diabetes Diagnosis | Model including  MetS Severity* |  | Model including  No-Glucose MetS Severity* |  | Model Including  ATP-III MetS* |  | Model including Number of  ATP-III MetS Components* |
| --- | --- | --- | --- | --- | --- | --- | --- |
|  | Hazard Ratio  (95% CI) |  | Hazard Ratio  (95% CI) |  | Hazard Ratio  (95% CI) |  | Hazard Ratio  (95% CI) |
|  |  |  |  |  |  |  |  |
| Time of Diabetes Diagnosis: |  |  |  |  |  |  |  |
|  |  |  |  |  |  |  |  |
| Visit 1 (n=536) | 1.24 (1.14, 1.35) |  | 1.33 (1.11, 1.58) |  | 1.38 (0.97, 1.97) |  | 1.13 (1.02, 1.26) |
|  |  |  |  |  |  |  |  |
| Visit 2 (n=372) | 1.34 (1.19, 1.52) |  | 1.61 (1.22, 2.11) |  | 3.19 (1.46, 6.96) |  | 1.41 (1.16, 1.71) |
|  |  |  |  |  |  |  |  |
| Visit 3 (n=255) | 1.40 (1.02, 1.92) |  | 1.59 (1.07, 2.36) |  | 2.07 (0.92, 4.67) |  | 1.37 (1.07, 1.76) |
|  |  |  |  |  |  |  |  |
| Visit 4 (n = 233) | 1.22 (0.91, 1.64) |  | 1.38 (0.95, 2.00) |  | 0.94 (0.40, 2.22) |  | 1.32 (0.95, 1.83) |
|  |  |  |  |  |  |  |  |
| *Interaction p-value*  *(Time of Diagnosis x MetS (Severity))* | *0.6840* |  | *0.6296* |  | *0.1409* |  | *0.1712* |
|  |  |  |  |  |  |  |  |

* Adjusted for baseline age, race, and sex

Table S3: Models of Standard MetS Severity in Individuals with Baseline Diabetes on Incident CHD

with and without inclusion of HbA1c at Visit 2

| Model Covariate | Model including  MetS Severity, age, sex, race | |  | Model including  MetS Severity, age, sex, race  and HbA1c at Visit 2 | |
| --- | --- | --- | --- | --- | --- |
|  | Hazard Ratio (95% CI) | p-value |  | Hazard Ratio (95% CI) | p-value |
|  |  |  |  |  |  |
| Standard (5-component)  MetS severity | 1.24 (1.13, 1.36) | < 0.001 |  | 1.21 (1.09, 1.34) | < 0.001 |
|  |  |  |  |  |  |
| No-glucose MetS severity | 1.34 (1.12, 1.60) | 0.001 |  | 1.25 (1.04, 1.51) | 0.020 |
|  |  |  |  |  |  |
